# Efficacy and safety of the Chinese herbal formula Hewei Jiangni recipe for NERD with cold-heat complex syndrome: study protocol for a double-blinded randomized controlled trial

**DOI:** 10.1186/s13063-021-05471-7

**Published:** 2021-08-18

**Authors:** Xiaosi Zhang, Yuan Cheng, Xiaohong Li, Xiang Tan, Lei Shi, Xiaojun Shi, Xiancui Zhang, Chun’e Xie, Junxiang Li

**Affiliations:** 1 Graduate School, Beijing University of Chinese Medicine, No. 11, North Third Ring East Road, Chaoyang, 100029 District. Beijing China; 2 Department of Gastroenterology, Dongfang Hospital, Beijing University of Chinese Medicine, No. 6 fangxingyuan Fengtai District, Beijing, 100078 China; 3 Department of Gastroenterology, Shenzhen Traditional Chinese Medicine Hospital, The Fourth Clinical Medical College of Guangzhou University of Chinese Medicine, Shenzhen, China

**Keywords:** Nonerosive gastroesophageal reflux disease, Randomized controlled trial, Hewei Jiangni recipe, Chinese herbal medicine, Study protocol

## Abstract

**Background:**

Proton pump inhibitor (PPI) is effective for the treatment of nonerosive gastroesophageal reflux (NERD), but long-term use of PPI is prone to have complications and recurrence after withdrawal. Traditional Chinese medicine (TCM) can relieve the symptoms of reflux and improve the quality of life. The purpose of this study is to evaluate the safety and efficacy of Hewei Jiangni recipe (HWJNR) in the treatment of NERD with cold-heat complex syndrome, and clarify the mechanism of HWJNR on NERD based on the correlation analysis of intestinal flora and metabolites.

**Methods:**

This is a single-center, randomized controlled, double-blind, placebo-controlled clinical trial in which 72 eligible participants with NERD and TCM syndrome of intermingled heat and cold will be randomly allocated in the ratio of 1:1 to two groups: TCM group and western medicine group. The TCM group will receive HWJNR with omeprazole enteric-coated tablets placebo, while the western medicine group will receive omeprazole enteric-coated tablets with HWJNR placebo. Each group will be treated for 8 weeks. The primary outcome is the score of gastroesophageal reflux disease (GERD) health-related quality of life questionnaire (GERD-Q). Secondary outcomes include SF-36 quality of life scale (SF-36), patient-reported outcomes (PRO) self-rating scale score, syndrome score of TCM, and adverse events. Mechanistic outcome is the correlation analysis of intestinal flora and metabolites from healthy individuals and NERD participants before and after the treatment respectively.

**Discussion:**

The goal of this trial is to investigate the efficacy and safety of HWJNR in the treatment of NERD with cold-heat complex syndrome, and to study the composition structure and metabolite expression profile of intestinal flora in patients with NERD through 16SrRNA sequencing and metabolomic correlation analysis of fecal flora, which makes us identify the dominant links of treatment and reveal the potential mechanism of HWJNR.

ChiCTR2000041225. Registered on 22 December 2020

## Administrative information

Note: the numbers in curly brackets in this protocol refer to Standard Protocol Items: Recommendations for Interventional Trials (SPIRIT) checklist item numbers. The order of the items has been modified to group similar items (http://www.equator-network.org/reporting-guidelines/spirit-2013-statement-defining-standard-protocol-items-for-clinical-trials/).

**Table:** 

Title {1}	Efficacy and safety of the Chinese herbal formula Hewei Jiangni recipe for NERD with cold-heat complex syndrome: study protocol for a double-blinded randomized controlled trial
Trial registration {2a and 2b}	ID: ChiCTR2000041225, registered on 22 December 2020. http://www.chictr.org.cn/showproj.aspx?proj = 66217
Protocol version {3}	Version 2.0, 11 November 2020
Funding {4}	This work was supported by National Natural Science Foundation of China (81803907) and Fundamental Research Funds for the Central Universities (No. 2020-JYB-ZDGG-128)
Author details {5}	Zhang Xiaosi^1,2^, Cheng Yuan^1,2^, Tan Xiang^1,2^, Li Xiaohong^2^, Shi Lei^3^, Shi Xiaojun^1,2^, Zhang Xiancui^1,2^, Xie Chune^2^, Li Junxiang ^2^ 1.Graduate School, Beijing University of Chinese Medicine, No. 11, North Third Ring East Road, Chaoyang District, Beijing 100029, China 2.Department of Gastroenterology, Dongfang Hospital, Beijing University of Chinese Medicine, No. 6 fangxingyuan Fengtai District, Beijing 100078, China 3. School of Life Science, Beijing University of Chinese Medicine, Beijing 100029, China Corresponding author: Li Junxiang, MD; No. 6 fangxingyuan Fengtai District, Beijing; lijunxiang1226@126.com, 18058335135

## Introduction

### Background {6-7}

Nonerosive gastroesophageal reflux (NERD) refers to the reflux disease with typical of gastroesophageal reflux symptoms and no mucosal injury under endoscope, also known as endoscopic negative reflux disease [1]. The incidence of gastroesophageal reflux disease (GERD) is increasing yearly based on the epidemiological investigation [2], which has a great impact on the quality of life. The incidence of NERD accounts for about 70% of GERD [3]. Therefore, the effective management of NERD is critical to human health.

Proton pump inhibitor (PPI) is the first-line drug for the treatment of reflux disease [4]. However, long-term use of PPI may lead to the deterioration of nocturnal symptoms and complications, including kidney injury, electrolyte abnormalities, and adrenal diseases [5–7]. The efficacy of PPI in the treatment of NERD is relatively rare compared with erosive esophagitis. Studies have shown that there is a tendency to relapse after drug withdrawal [8]. Due to repeated attacks and side effects of long-term acid suppression, NERD has increasingly become an important and thorny problem in the digestive system.

Vonoprazan, a potassium-competitive acid blocker (P-CAB), is a new type of medicine for the treatment of GERD launched in Japan. P-CABs are stable in acidic environments and exert more potent and prolonged acid-inhibitory effects than PPIs [9]. According to a systematic review, vonoprazan may have a better maintenance effect on GERD compared to PPI, but its precise function is not completely clear for NERD [10]. PPIs effect on acid reflux, but not the main underlying mechanism for GERD (transient lower esophageal sphincter relaxation, TLESR) [11]. Karim et al. believe that transoral incisionless fundoplication (TIF) is more effective than PPI therapy in eliminating troublesome regurgitation and extraesophageal symptoms of GERD [12]. A UK collaborative randomized trial showed that at least up to 12 months after laparoscopic fundoplication, the surgery significantly increased measures of health status in patients with GERD [13]. Surgical treatments have many advantages, but are prone to complications, especially esophageal perforation, which may outweigh any potential risk of long-term use of PPI. Surgical treatment may also face the problems of short duration of treatment and easy recurrence [14, 15].

From the perspective of traditional Chinese medicine (TCM), the stomach belongs to dryness and Yang soil, and majority of them belong to excess and heat, while the spleen belongs to dampness and Yin soil, and most of them belongs to deficiency and cold. According to the clinical observation and research of TCM syndromes, more and more patients with NERD are characterized by mixed syndrome of intermingled heat and cold. This is inseparable from the changes in people’s living standards and dietary structure in modern society. They not only suffer from acid reflux, heartburn, dry mouth, bitterness, and other syndromes of excess heat, but also syndrome of deficiency cold, such as aversion to cold and loose stools. The main features of cold-heat complex syndrome are as follows: (1) burning discomfort behind the sternum or epigastric part; (2) acid regurgitation or vomiting clear water; (3) dull pain in the epigastrium, like warming and pressing. The general principle of TCM treatment is pungent dispersing and bitter descending, harmonizing stomach, and descending adverse qi [16].

Hewei Jiangni recipe (HWJNR) was created by Professor Junxiang Li of Dongfang Hospital, Beijing University of Chinese Medicine on the basis of inheriting “Tongjiang theory” in the treatment of spleen and stomach diseases. Table 1 lists the detailed formula. This prescription was based on the in-hospital preparation (“Hejiang capsule” approval number: Beijing Pharmaceutical Z20160002) of Dongfang Hospital to treat NERD patients with cold-heat complex syndrome specifically. Our research group conducted *an observational study* in 2002, which showed that HWJNR could reduce heartburn, acid regurgitation, and other clinical symptoms. The recurrence after 12 weeks withdrawal was significantly less than that in western medicine group. At the same time, studies in mechanism have obtained good progress [17, 18].

**Table 1:** Formula of HWJNR

Pinyin name	Latin name	Family name
Huangqin	Radix Scutellariae	Labiatae
Huanglian	Coptidis Rhizoma	Ranunculaceae
Qingbanxia	Pinelliae Rhizoma	Araceae
Ganjiang	Zingiber officinale Rosc.	Zingiberaceae
Zhebeimu	Fritillaria thunbergii Mip.	Liliaceae
Pugongying	Taraxacum mongolicum Hand.-Mazz.	Compositae
Longdancao	Gentianae Radix et Rhizoma	Gentianaceae
Zhishi	Citrus aurantium L.	Rutaceae
Quangualou	Trichosanthis Fructus	Cucurbitaceae
Zhigancao	Glycyrrhiza uralensis Fisch.	Leguminosae

Through the single-center, randomized, double-blinded, placebo-controlled clinical research, this trial aims to evaluate the efficacy and safety of HWJNR in patients with NERD (cold-heat complex syndrome). In addition, this trial intends to reveal the underlying pathogenesis by observing the effects of changes in intestinal bacteriocyte structure on metabolic ideotypes and functional metabolic small molecules.

## Methods/design

### Study design and setting {8-9}

This is a single-center, double-blinded, placebo-controlled, randomized controlled trial (RCT), which will be conducted in Dongfang Hospital, Beijing University of Chinese Medicine. The study is conducted in accordance with the Declaration of Helsinki (Edinburgh 2000 version). The final protocol (Version 2.0, 11 November 2020) of this trial has been approved by Ethical Committee of Dongfang Hospital, Beijing University of Chinese Medicine (Version number: JDF-IRB-2020033602). In addition, this trial has been registered in the Chinese Clinical Trial Registry (No. ChiCTR2000041225, registered on 22 December 2020).

**Fig. 1:**
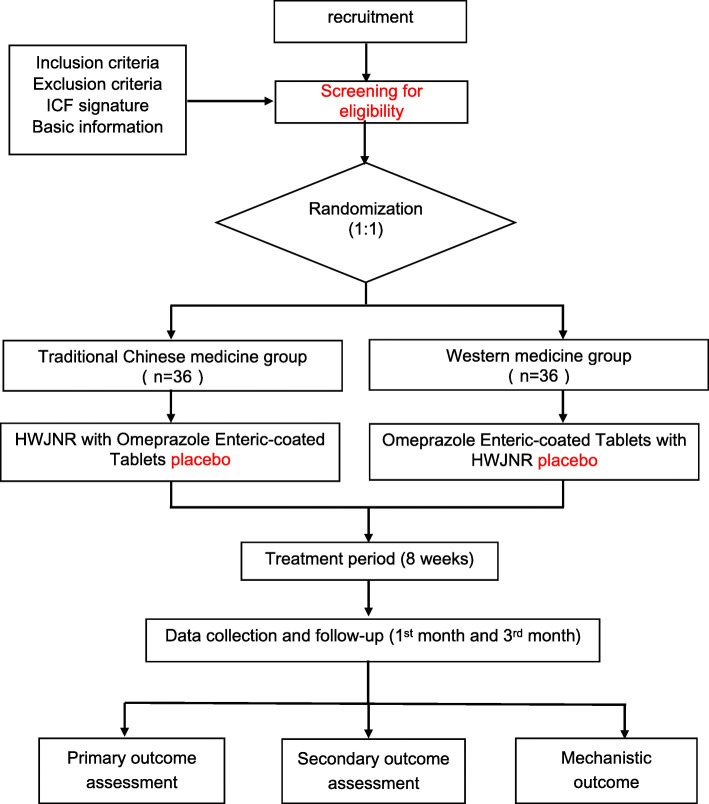
Schematic process of the study

We aim to enroll 72 patients with NERD over a 2-year period (Date from November 2020 to December 2022) from the department of gastroenterology in Dongfang Hospital, Beijing University of Chinese Medicine. Eligible patients will be randomized at a ratio of 1:1 to either TCM group (HWJNR with omeprazole enteric-coated tablets placebo) or western medicine group (omeprazole enteric-coated tablets with HWJNR placebo) for 8 weeks treatment. At the end of this trial, the primary outcome (GERD-Q) and the secondary outcome (SF-36, PRO, syndrome score of TCM) will be evaluated. We will obtain peripheral venous blood, urine, and stool from NERD patients before and after treatment. This study involves 3 site visits (week 0, week 4, and week 8) and 10 call visits (weekly call during the treatment period and follow-up at month 1 and month 3). The process of the study is schematically shown in Fig. 1. And we will additionally recruit 10 healthy controls.

### Participants

We aim to recruit patients who have typical gastroesophageal reflux symptoms (such as acid reflux and heartburn), or who do not have typical gastroesophageal reflux symptoms, but have 24-h impedance-pH monitoring showing that they are related to gastroesophageal reflux. The symptoms above are not less than three months, and the gastroscopy show nonerosive gastroesophageal reflux. The inclusion criteria of TCM refers to the diagnosis of cold-heat complex syndrome in the “consensus on Integrated traditional Chinese and Western Medicine diagnosis and treatment of gastroesophageal reflux Disease” issued by the Digestive Professional Committee of the Chinese Society of Integrated traditional Chinese and Western Medicine in 2017 (Table 2). The inclusion criteria of western medicine is based on the consensus of experts on gastroesophageal reflux disease issued in 2014 (Table 3). And we will also recruit 10 healthy subjects for control.

**Table 2:** Diagnostic criteria for cold-heat complex syndrome of NERD

Category	Symptoms or signs
Main symptom	Burning discomfort in the retrosternal or epigastric region. Anti-acid or spitting clear water. Dull pain in the epigastric stomach, like warming, and pressing. Fasting stomachache, relieved by eating.
Minor symptoms	Loss of appetite; Fatigue; Thin stools; Lukewarm hands and feet.
Tongue	Red tongue, White fur
Pulse condition	Weak pulse

**Table 3:** Inclusion criteria of NERD

Those who have the typical clinical symptoms of NERD, such as heartburn and reflux, persist or recur for more than 3 months, and the above symptoms have a negative impact on the quality of life of the patients. Those who have atypical or extraesophageal symptoms, such as epigastric pain, abdominal distension, belching, non-cardiogenic chest pain, pharyngitis, cough, asthma, or foreign body sensation in the throat, which persist or recur for more than 3 months, the above symptoms have a negative impact on the patient’s quality of life: mild symptoms ≥ 2 days in a week, or moderate and severe symptoms ≥ 1 days in a week. Reflux diagnostic questionnaire (RDQ) scale symptom score ≥ 12, which is for diagnosis of reflux diseases in the China gastroesophageal reflux Research Cooperation Group. Those who have no esophageal mucosal lesions and Barrett esophageal manifestations by gastroscopy. Tweny-four-hour esophageal impedance-PH monitoring: impedance records showed reflux events. At the same time, symptom association probability (SAP) is used to determine the relationship between symptoms and reflux. If SAP > 95%, symptoms will be considered related to reflux.	

Diagnostic criteria: 1&3&4 or 2&4&5

The main symptom 1 or 2 is necessary. Patients with two main symptoms + either of the other one or two minor symptoms + appropriate tongue and pulse condition can be diagnosed as cold-heat complex syndrome

### Eligibility criteria {10}

#### Inclusion criteria for subjects

1. Patients meet the diagnostic criteria of NERD in guide and TCM with cold-heat complex syndrome at the same time.
2. 18–65 years old.
3. Those who did not receive similar drugs (PPI, H2 receptor antagonist, gastric mucosal protective agent, etc.) within 2 weeks before the trial.
4. Voluntarily participated in this study and signed the informed consent form (ICF).

#### Exclusion criteria

1. Patients with reflux esophagitis, reflux hypersensitivity (RH), or Barrett esophagus (BE).
2. Patients with a disease history of upper gastrointestinal bleeding or surgery, esophageal stricture, esophageal and gastric tumors, and other organic diseases.
3. Patients with serious primary diseases, such as cardiovascular, cerebrovascular, liver, kidney, and hematopoietic systematic diseases.
4. Taking other drugs or treatments related to NERD (PPI, H2 receptor antagonist, gastric mucosal protective agent, etc.) within 2 weeks.
5. Women who are breastfeeding, during pregnancy, or preparing for pregnancy.
6. Patients with anxiety or depression.
7. Patients with a history of mental ill or neurological diseases or language disorders.
8. Allergic constitution or being allergic to the known ingredients of the drug in the study.
9. Currently participating in other clinical trials.

#### Inclusion criteria for healthy subjects

1. Those who have no typical clinical symptoms of NERD such as acid regurgitation, heartburn, and reflux.
2. The score of GERD-Q scale < 8 points.
3. 18–65 years old.
4. Those who voluntarily participate in this study and sign the ICF.

#### Exclusion criteria for healthy subjects

1. Those who have been diagnosed as esophageal diseases such as NERD, RH, and BE.
2. Patients with organic lesions such as upper gastrointestinal bleeding or operation, esophageal stricture, esophageal, and gastric tumors.
3. Patients with serious primary diseases such as cardiovascular, cerebrovascular, liver, kidney, and hematopoietic system.
4. Women who are breastfeeding, pregnant, or preparing for pregnancy.
5. Patients with anxiety or depression.
6. Those who with a history of mental or neurological disorders/diseases.
7. Patients who have participated in any other clinical trials within 3 months.

### Sample size {14}

According to clinical experience, the effective rate of NERD in the TCM group was P1 = 92.0%. The NERD effective rate of the western medicine group was P2 = 61.2% [(two-sided type I error rate of 0.05, *α* = 0.05); (one-sided type I error rate of 0.20, *β* = 0.20)]. The sample size is calculated by the following formula:

$$ n=\frac{2\overline{p}\overline{q}{\left({Z}_{\alpha }+{Z}_{\beta}\right)}^2}{{\left(P1-P2\right)}^2} $$

*n*1 = *n*2 = 2 × 0.766 × 0.234 × 2.8^2^ ÷ 0.308^2^ = 30. Taking approximate dropouts of 20% into account, the number of participants recruited was estimated to be 36 per group. Therefore, a total of 72 patients will be recruited in this trial. And we will additionally recruit 10 healthy controls.

### Recruitment {15}

All the cases will be from outpatients of digestive department in Dongfang hospital, Beijing University of Chinese Medicine, who were clinically diagnosed as NERD, and dialectically belonged to the syndrome of intermingled heat and cold.

### Allocation {16}

The drugs are allocated according to the random coded sequence of the research center and the number of cases. We will designate a research drug administrator. The drug will be distributed by the administrator according to the method of randomization and blinding and registered in the “Clinical Research Drug use record form” after the researchers screen the qualified subjects and wrote the research medical records with informed consent.

### Randomization and blinding {16.17}

The research drugs will be randomly coded according to the randomized scheme of clinical research in Xiyuan Hospital of the Academy of TCM. (1) The method of random allocation: using SAS software to generate random sequences, randomly grouping, and coding drugs; (2) the distribution scheme of hiding and implementing: the sealed envelope method will be used to hide the random sequences.

Blindness will be set for the implementers (doctors) of the study and the subjects (patients). The random code of the drug used will be the unique identification code of the subjects. Each coded drug will be accompanied by an emergency treatment plan in the letter. If emergencies occur in the treatment period, implementers need to know what kind of treatment the patient is receiving. The letter shall be opened in the presence of at least two researchers and collected together with the case report form (CRF) after the end of the trial. Blind leakage or emergency letter opening should not exceed 20% before the end of the trial.

### Consent and assent {26a,27}

Implementers will explain the purpose of the study and benefits/risks to participate who volunteer to join the research group and sign an ICF. The main investigator will be responsible for obtaining informed consent. All the medical records will be kept in Dongfang hospital. It will be allowed to access medical records of patients for researchers, research authorities, and ethics committees. The specimens collected in the test will be destroyed after use. Any public report on the results of this study will not disclose the personal information of the patients.

### Interventions {11}

#### Intervention description {11a}

TCM group was given HWJNR with omeprazole enteric-coated tablets placebo; western medicine group was given omeprazole enteric-coated tablets with HWJNR placebo. The course of treatment is set for 8 weeks. Medication method and dose were as follows: omeprazole enteric-coated tablets/placebo: 20 mg, once a day, half an hour before breakfast; HWJNR/placebo: twice a day, one bag at a time, half an hour after breakfast and dinner. For production of formula placebo: the TCM placebo is made by Beijing Kangrentang Pharmaceutical Co., Ltd., according to the preparation process of HWJNR. The shape, color, gas, and taste will be similar to HWJNR after adding the same kind and equal amount of excipients. Finally, 5% of the original unit treatment drug is added to correct color and taste.

### Adverse reaction {11b}

The change or discontinuation of the intervention will be determined by the performance of the participants after taking the drug. The researcher will truthfully reflect the changes of the condition during the study period. Our first priority is to protect the safety of the subjects. If the symptoms of gastroesophageal reflux such as reflux and heartburn have not been effectively relieved, hydrotalcite chewable tablets (Daxi) will be used for temporary treatment, and the total dosage of hydrotalcite chewable tablets in the two groups will be counted at the end of the trial.

### Compliance {11c}

We will issue CRFs to all subjects and supervise patients to fill CRFs every day to test compliance. Compliance with medication% = [actual dose/(specified daily dose × days)] × 100%. We will record the patient’s symptoms every day, and finally score according to CRFs. Messages will be sent through WeChat or by phone to remind the patients of the follow-up. Results of physical examinations will be explained at each visit. All the tests and drug fees during the trial will be covered.

### Combined use of drugs {11d}

During the study period, drugs with the same efficacy as the study drugs or related to the treatment of NERD will be prohibited to reduce intervention. If the subjects take drugs to control chronic diseases, such as hypertension and diabetes, they should truthfully and detailedly record the medication in the trial, including drug name (or other treatment name), dosage, frequency, and time of use.

### Outcomes {12}

#### Primary outcome measurements

##### GERD-Q scale score

GERD-Q scale is the most recognized and widely used special scale for GERD diagnosis in the world. In addition to diagnosing GERD, it can also evaluate the impact of GERD on quality of life and monitor the effect of treatment with high accuracy. A trained evaluator will record the evaluation at baseline, week 4, and week 8, fill out the GERD-Q questionnaire, and calculate the total score, frequency score, and degree score of GERD-Q symptoms. In the study, the score of GERD-Q scale after 8 weeks treatment will be used as the main outcome index.

#### Secondary outcome measurements

1. Quality of life score: According to the Chinese version of SF-36 health survey [19], the quality of life will be evaluated from 9 dimensions: physical function, physical function, physical pain, general health, vitality, social function, emotional function, and mental health. All the indexes will be recorded at baseline, week 4, and week 8.
2. Patient-reported outcomes (PRO) self-rating scale score: There will be a diary card for patients to fill in every day. The researchers will calculate the PRO self-rating scale score according to the patient’s diary card at baseline, week 4, and week 8.
3. TCM clinical syndrome score: According to the diagnostic criteria of cold-heat complex syndrome, the main and minor symptoms, tongue, and pulse will be graded and scored by using a unified table. All the indexes will be recorded at baseline, week 4, and week 8. According to the referred Guidance Principle of Clinical Study on New Drug of Traditional Chinese Medicine [20], all symptoms will be divided into four grades: none, mild, moderate, and severe, with 0, 2, 4, and 6 points in the main symptoms, and 0, 1, 2, and 3 points in the minor symptoms. In addition, the tongue and pulse will be divided into normal and abnormal grades, with 0 and 2 points.

The clinical efficacy will be evaluated as follows:

- Clinical recovery: the symptoms disappeared or syndrome score decreased by ≥ 95% from the baseline.
- Marked efficacy: the syndrome score decreased by ≥ 70%, but < 95% from the baseline.
- Effective: the syndrome score decreased by ≥ 30%, but < 70% from the baseline.
- Ineffective: the symptoms and signs were not significantly improved, or even aggravated, and the syndrome score decreased by < 30% from the baseline.
- Aggravation: the syndrome score after treatment is higher than that before treatment.

TCM syndrome efficacy rate = (clinical recovery + marked efficacy + efficacy) cases/a total number of cases × 100%.

(4) Mechanistic outcome: The samples will be used to study the composition structure and metabolite expression profile of intestinal flora in NERD patients through 16s rRNA sequencing and metabonomic association analysis in TCM group, western medicine group at baseline and week 8, and in normal subjects at baseline. By analyzing the correlation between a variety of differential flora and differential metabolites, we will demonstrate the effects of changes in the structure of intestinal flora on metabolic phenotypes and functional metabolic small molecules. This experiment attempts to screen some key strains through visual tools, further explore their mechanism in the pathogenesis of NERD, identify the advantages of treatment, and reveal the potential mechanism of HWJNR. The determination of intestinal flora and metabolites will be performed by Shanghai OE Biotech Co., Ltd (Shanghai, China).
(5) Follow-up recurrence index: The number and percentage of the subjects with non-medication, maintenance medication, intermittent medication, and on-demand medication will be recorded at month 1 and month 3 respectively.

### Safety outcomes

Participants will undergo laboratory tests at baseline and week 4, including liver and kidney function (ALT, AST, Scr, BUN), blood routine test, urine routine test, and stool routine test. Other tests include an electrocardiograph examination.

### Participant timeline {13}

Table 4 shows the schedule of enrollment, treatment, and assessment.

**Table 4:**
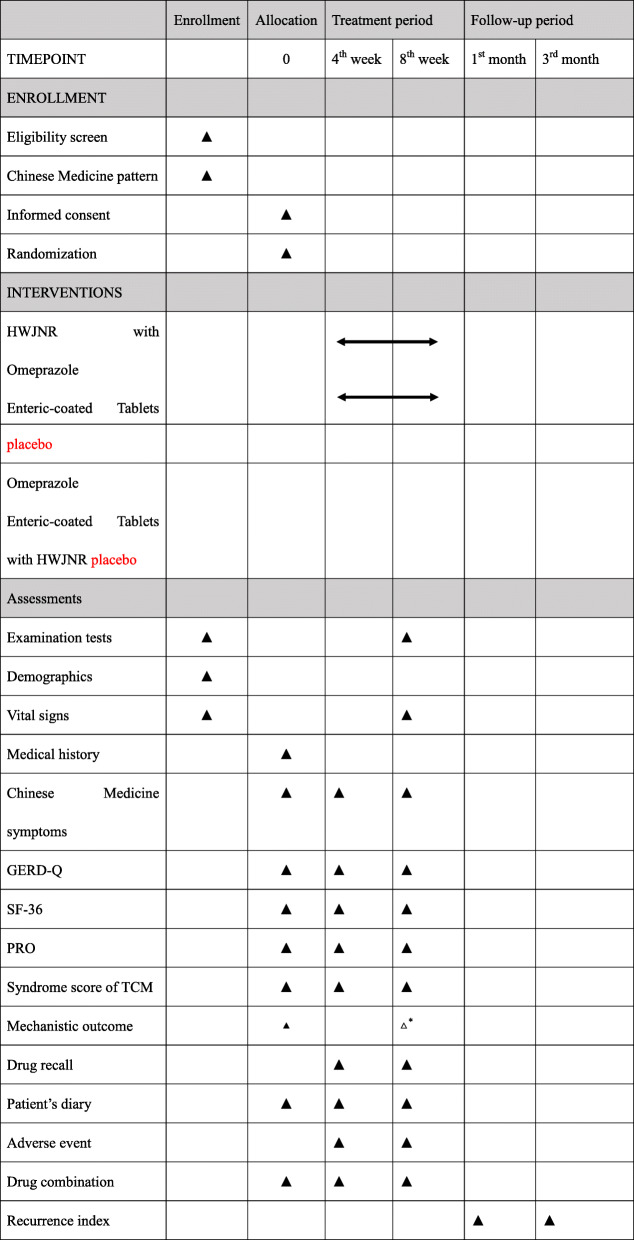
Schedule of enrollment, treatment, and assessment

*The mechanistic outcome will be analyzed in experimental group (TCM group and western medicine group) at the baseline and week 8, and in normal subjects at baseline

### Biological specimens {26b,33}

We will collect human samples including blood, urine, and feces. These samples will be used for blood routine test, urine routine test, stool routine test, and liver and kidney function test. The feces will additionally be used to detect the relationship between fecal flora and metabolites. All specimens will be destroyed after examinations.

#### Data collection and management {18,19}

##### Recording requirements of the CRFs

1. All records will be collected and screened by investigators, who has been trained and qualified. Researchers must fill in the research forms in a timely, accurate, complete, and standardized manner based on the relevant regulations and instructions. All data will be collected, including the participant retention and complete follow-up. If there is any doubt about the data, our investigator will send an “electronic data inquiry” to the researcher. The researcher should reply as soon as possible. The main researchers will be responsible for the authenticity of the research data.
2. The laboratory test must be pasted on the “Research Medical record.” The laboratory test or description recorded in the CRFs should be checked correctly with the original test report. Once the CRFs are complete, the original record will not be changed, even if any modifications are made. The completed CRFs will be reviewed by a clinical inspector.
3. The data of laboratory examination items outside the normal range should be confirmed by the researchers and reexamined. During the course of the trial, abnormal examination items with clinical significance should be recorded and followed up based on adverse events.
4. All data will be kept by a special person.

##### Selection of dataset

1. Full analysis set (FAS): FAS refers to the intention-to-treat (ITT) analysis of all patients who have been randomized into groups and assigned to random numbers (called willing treatment groups). Errors that do not meet the inclusion criteria or exclusion criteria are excluded.
2. Per-protocol set (PPS): PPS refers to the statistical analysis of all cases that is in accordance with the clinical observation plan (Compliance with medication ranging from 80 to 120% will be eligible for the protocol analysis set). Subjects will not take prohibited drugs during the observation period, and completed the CRF requirements.
3. Safety set (SS): Safety data should include all subjects who have received at least one treatment and at least one safety assessment after randomization.

### Statistical analysis {20}

Efficacy analysis will be performed based on both FAS and PPS patients. Safety analysis includes all randomized participants who take at least one bag of HWJNR and have data for safety assessment. Comparing the healthy control group with the traditional Chinese medicine group, we will study the composition of the intestinal flora and the expression of metabolites in patients with NERD and the healthy group. And it will also reveal the mechanism of action and advantages of HWJNR in the treatment of NERD patients.

Changes from baseline to post-treatment in GERD-Q, SF-36, PRO, and syndrome score of TCM will be analyzed using analysis of covariance with associated 95% confidence intervals. All the analysis results will be generated by SPSS 25.0 software. Repeated measurement data are expressed by mean ± standard deviation, intra-group comparison is performed by analysis of variance of repeated measurement data, and inter-group comparison is by multivariate analysis of variance (MANOVA). *P* ≤ 0.05 indicates that the difference is statistically significant. Unilateral test will be used in all statistical tests, and the significance level *P* ≤ 0.05. The arithmetic mean, standard deviation, median, minimum, and maximum will be calculated according to the data distribution. Counting data will be used to calculate the number and percentage of cases.

### Data monitoring {5d, 21, 23}

Independent clinicians and biostatisticians with extensive research experience in clinical trials will serve as the Data and Safety Monitoring Committee. Two independent data administrators perform dual data entry and proofreading. The primary researchers, data administrators, and biostatisticians will check, confirm, and lock the database before data processing. Only data administrators and biostatisticians will have access to the final data. The Ethics Committee of Dongfang Hospital will conduct annual audits and supervise protocol, adverse effects reporting, and interim results. Researchers will submit research progress reports within 1 month before the deadline.

### Adverse event reporting and harms {22,30}

An adverse event (AE) refers to any adverse medical event that occurs to the patient or clinical investigation object during the period of management of drugs, but it does not necessarily have a causal relationship with this treatment. According to the standards developed by the Adverse Drug reaction Supervision Center of the Ministry of Health, we will classify them according to the five-level classification of “affirmative, probable, probable, suspicious and impossible.” The former four will be taken together to study the AEs of drugs, and the incidence of AEs will be calculated accordingly (Table 5).

**Table 5:** Adverse drug reaction supervision center of the Ministry of Health. Evaluation criteria for the relationship between AE and research drug use

Judgment index	Judgment result				
	Definitely	Probably	Probably	Suspiciously	Impossible
1. Whether there is a reasonable relationship between the time of starting medication and the time of suspicious occurrence.	+	+	+	+	+
2. Whether the suspected ADR (ADR) conforms to the known ADR type of the drug	+	+	+	−	−
3. Whether the suspected ADR can be explained by the patient's pathological condition, combined medication, therapy or previous therapy.	−	−	±	±	+
4. It is doubtful whether the withdrawal or reduced dose of ADR alleviates or disappears	+	+	±	±	−
5. Does the same reaction occur again after re-exposure to suspicious drugs	+	?	?	?	-

Description: + for positive, − for negative, ± for positive or negative, ? indicates that the situation is unknown

Before the trial, no AE has been reported in the clinical application of HWJNR. The possible AEs of omeprazole enteric-coated tablets include diarrhea, headache, nausea, abdominal pain, flatulence and constipation, and occasional increase of serum aminotransferase (ALT, AST), rash, dizziness, and so on. These AEs are usually mild and can disappear automatically, which have nothing to do with the dose. Since the omeprazole enteric-coated tablets used in this study belong to the routine treatment of NERD, these AEs may also occur even if the subjects do not participate in the clinical study. During the study, we will closely monitor AEs and take measures to prevent them. Once the emergencies related to the study occur, the research assistant will record the detailed information including symptoms, signs, severity, start date, duration, lab results, intervention, and the results of adverse events at each visit. And the subjects will have the right to receive corresponding compensation.

Serious adverse event (SAE) refers to any adverse medical accident that occurs at any dose, including death, life-threatening conditions, the need for hospitalization or prolongation of existing hospitalization, persistent or significant disability/incapacity, or congenital anomaly/birth defect. Once SAEs occur, the events must be reported to the principal investigator and the Ethics Committee and the Beijing Food and Drug Administration within 24 h. The principal investigator has the power to terminate the trial if necessary.

## Discussion

The study is a single-center, randomized controlled, double-blind, placebo-controlled clinical trial for HWJNR to evaluate the efficacy and safety in the treatment of NERD. NERD is characterized by the presence of typical GERD symptoms associated with pathological acid reflux, including heartburn and regurgitation, but the absence of esophageal erosion [21, 22]. NERD presents in approximately 70% of patients with GERD, which has a great impact on life quality [3]. The high incidence of NERD and the widespread use of PPIs in clinical practice mean that the problems of complications and recurrence after drug withdrawal are increasingly common. TCM has a significant role in alleviating symptoms and maximizing quality of life of NERD patients. Hence, GERD-Q, SF-36, PRO, and syndrome score of TCM will be selected as therapeutic indicators, since they are correlated to the patients’ symptoms and quality of life.

HWJNR is mainly made up of Huangqin, Huanglian, Qingbanxia, Ganjiang, and other ten herbs. In our previous research, HPLC analysis was used to measure the contents of HWJNR. The results showed that the repeatability and stability of these main ingredients of HWJNR are good, and the quality is controllable [18]. Previous clinical studies have shown that HWJNR is safe and effective in NERD.

The factors that affect NERD have not yet been clarified [23]. There are several hypotheses about the etiology of NERD, such as esophageal visceral hypersensitivity, sustained esophageal contractions, and abnormal tissue resistance [24]. Recently, studies focus on abnormal microbial-brain-gut interaction and low-level inflammation of GERD mediated by hypoxia-inducible factor [25, 26]. We also expect to have a deeper understanding of the dominant links of treatment, reveal the potential mechanism of HWJNR, and study the composition structure and metabolite expression profile of intestinal flora in patients with NERD.

We aim to provide new and high-quality evidence in HWJNR for patients with NERD (cold-heat complex syndrome) through the clinical trial. The research results are expected to help formulate relevant clinical programs and corresponding guidelines. However, this study is a small sample size clinical study conducted in Beijing of China. Therefore, it is necessary to conduct multicenter clinical trials in further research.

## Trial status

This updated version of the trial protocol (version 2.0, 11 November 2020) has been reviewed and approved by the Ethics Review Committee of Dongfang on 7 December 2020. All relevant researchers have completed the standardization training concerning this trial in December 2020. The first patient was recruited in the trial on 15 January 2021. By the time our manuscript was submitted, we had recruited 10 volunteers. Recruitment is scheduled to be completed in December 2022.

## Abbreviations

AE: Adverse event
BE: Barrett esophagus
CRF: Case Report Form
FAS: Full Analysis Set
GERD: Gastroesophageal reflux disease
GERD-Q: Gastroesophageal reflux disease health-related quality of life questionnaire
HWJNR: Hewei Jiangni recipe
ICF: Informed Consent Form
ITT: Intention-to-treat
NERD: Nonerosive gastroesophageal reflux
MANOVA: Multivariate analysis of variance
PPI: Proton pump inhibitor
PPS: Per-protocol set
PRO: Patient-reported outcomes
P-CAB: Potassium-competitive acid blocker
RCT: Randomized controlled trial
RDQ: Reflux Diagnostic Questionnaire
RH: Reflux hypersensitivity
SPIRIT: Standard Protocol Items: Recommendations for Interventional Trials
SAE: Serious adverse event
SAP: Symptom association probability
SF-36: SF-36 quality of life scale
SS: Safety set
TCM: Traditional Chinese medicine
TIF: Transoral incisionless fundoplication
TLESR: Transient lower esophageal sphincter relaxation
